# Two Sides of the Same Coin: Sensation Seeking Fosters Both Resiliency and Tobacco and Alcohol Use Among 16-Year-Olds

**DOI:** 10.3389/ijph.2023.1604777

**Published:** 2023-05-30

**Authors:** Magdalena Jochimek, Mariusz Lipowski, Małgorzata Lipowska, Ariadna Beata Łada-Maśko

**Affiliations:** ^1^ Department of Psychology, Faculty of Physical Culture, Gdansk University of Physical Education and Sport, Gdańsk, Poland; ^2^ Institute of Psychology, Faculty of Social Sciences, University of Gdansk, Gdańsk, Poland

**Keywords:** adolescence, physical activity, risky behaviour, sensation seeking, resiliency

## Abstract

**Objectives:** Sensation seeking (SS) is associated with engaging in risk behaviors and it is also positively correlated with engaging in physical activity and building beneficial personality resources for coping processes. This study investigates the role of SS in building resiliency and the risk of tobacco and alcohol use.

**Methods:** A total of 649 adolescents, who either practice or do not practice sports, took part in this study. Participants completed a set of questionnaires which verify level of: SS, resiliency, tobacco and alcohol use.

**Results:** No statistically significant gender- or sports-related differences were observed on the tobacco and alcohol use, as well as for SS according to the ANOVA results. Furthermore, mediation analysis showed that the effect of SS on tobacco and alcohol use through resiliency was significant for the female PE and the male athlete group.

**Conclusion:** Higher influence of SS on resiliency was noted in the male athlete group, and in this case resiliency was a factor protecting against tobacco use. Engaging in sports fosters resiliency, the mechanisms underlying the development of resiliency seem to be aided by SS.

## Introduction

Experimenting with risky behaviors is considered a normative behavior in adolescence [1]. However, it also makes teenagers approach situations that pose health risks [2]. Tobacco use usually starts in adolescence, and alcohol is the most commonly used psychoactive substance among adolescents [3]. According to the interactive model, explaining the problem behavior of adolescents, the interaction of environmental and individual factors is responsible for undertaking and continuing to engage in risky behaviors (Jessor, Jessor, 1990). The main environmental risk factors include socioeconomic factors, normative beliefs in the social environment, as well as normative conflict between parents and peers. The tendency to take risks is also related to individual dispositions [4], and engaging in risky behaviors is especially positively correlated with temperamental variables, such as impulsivity [5–7], lower sensory sensitivity, emotional reactivity and endurance, higher activity [8], and high levels of sensation seeking (SS) [9–11].

### Sensation Seeking as a Risk Factor

Sensation seeking (SS) is defined as “the seeking of varied, novel, complex and intense situations and experiences and the willingness to take physical, social, legal, and financial risks for the sake of such experience” ([12] p. 10]). Understood in this way, temperamental traits arepositively correlated with drug use [13, 14], engaging in risky sexual behavior [15] and careless driving [16]. On the basis of this characteristic, the risk of gambling addiction can also be anticipated [17]. Prior research has indicated that the highest levels of this trait (independent of gender) are recorded between 16–19 years of age [12, 18]. High levels of SS encourage experimenting with and repeating risky behaviors [10, 11, 12, 19]. This relationship is visible with regard to tobacco use [20–23] and alcohol use [22, 24–27].

It should be mentioned that both risk factors and factors protecting against problem behaviors interact with each other. For this reason, in this study, we consider the influence of sensation seeking on the tendency towards tobacco and alcohol use, as risky behaviors typical of the period of adolescence. It should be noted that sensation seeking is also associated with behaviors that are constructive from the individual’s perspective, such as undertaking physical activity, and may contribute to building beneficial personality resources for coping processes.

### Sensation Seeking, Physical Activity and Risk Behaviors

Research indicates that people engaging in risky and extreme sports [28] have a higher level of sensation seeking. It is also worth noting that youths’ thirst for SS may be quenched by physical activity [29]. Teenagers who practice sport have higher levels of SS than those who do not [30, 31].

The association of SS with cigarette smoking and alcohol consumption should also be highlighted. In boys, SS is associated with both higher levels of physical activity and with smoking cigarettes [32]. According to the model proposed by Audrain-McGovern and Rodriguez [33], physical activity (PA) reduces the probability of smoking cigarettes in adolescents [34, 35]. The research also confirmed that among people who are engaged in PA cigarette smoking is not a very widespread behavior [36, 37].

Mastroleo et al. [30] indicate that SS is a mediator of the relationship between levels of sporting achievement and alcohol use. In addition, SS is more related to episodes of drinking large amounts of alcohol among college students who are athletes compared with those who are non-athletes [38]. It ought to be added that higher involvement in physical activity is positively correlated with alcohol use among males [39, 40] but not females [41]. However, other studies suggest that physical activity may also be negatively associated with the risk of alcohol use in girls [36, 42]. Therefore, research on the impact of SS on the risk behavior of athletes is an area requiring further empirical exploration.

### Sensation Seeking and Resiliency

As mentioned earlier, higher levels of SS are not only related to developmental risks; it can also have a beneficial influence on an individual’s functioning. Thus, this temperamental variable may foster the development of a protective personality factor—resiliency—an individual disposition that activates and supports the process of coping with traumatic events and everyday stress [43]. McKay and Skues [44] suggest that SS supports psychological resiliency both directly and indirectly by modifying the stress response and strengthening personal resources for coping with difficulties. Research shows that depending on the resiliency level continuum aspect of a teenager’s personality profile, they will exhibit symptoms characteristic of either internalizing (overcontrolled profile) or externalizing (undercontrolled profile) behaviors [45]. The resilient profile is associated with a lack of psychopathological symptoms and the most flexible behavior, but it does not rule out experimentation (e.g., with alcohol) associated with a certain age. Research shows that higher levels of resiliency are associated with lower probabilities of engaging in risky behaviors; however, one of its dimensions—social competences—may act as a factor that makes it more likely to engage in such behaviors [46]. Thus, resiliency is a multifaceted construct, and it is possible to measure its influence in terms of its particular dimensions.

It should be added that psychological competencies associated with sport foster the development of resiliency [47], and athletes are characterized by higher levels of this personality feature [48–50]. Intensive sports training is associated with a lifestyle oriented towards achieving training goals and the greatest possible athletic success. Mastery of sport requires engagement, self-discipline, and persistence. The personality profile of an athlete can be examined from two perspectives. On the one hand, the specific sporting environment (e.g., other challenges, stressors, daily schedule, travelling for sport meets) may influence the development of certain dispositions, visible in the individual differences between young athletes and their peers. On the other hand, it is possible that individuals with a certain personality profile, one that predisposes them towards persistence and maintaining a demanding training routine, are more likely to engage in sport. The overlap of these two perspectives is the fact that individuals who are unable to adapt to a dynamically changing environment and the demands and challenges of their sport will remain at their current level or will have to quit. For this reason, analyzing the personality dispositions of athletes seems to be an interesting direction of research, especially in the context of risky behavior.

### Research Framework

In summary, sensation seeking seems to be a risk factor connected to tobacco and alcohol use, while resiliency is a protective factor against risky behavior. The level of sensation seeking thus seems to be a factor that fosters positive adaptation to unanticipated and stressful situations. McKay and Skues [44] have shown that sensation seeking fosters psychological resiliency. Moreover, a number of studies concerned with the importance of risky behaviors for health have been conducted on athletes in late adolescence and developing adulthood, while fewer analyses on this matter have been conducted with adolescents who start intense sports training [30].

Health Behavior in School-aged Children (HBSC) research indicates the existence of gender differences—boys are more likely to consume larger amounts of alcohol, especially after reaching the age of 15. However, the gender differences in tobacco use are diminishing [51]. Because the mechanisms of the influence of temperamental variables on risky behaviors are different in girls and boys, the gender of participants should be taken into account when analyzing this phenomenon.

**FIGURE 1:**
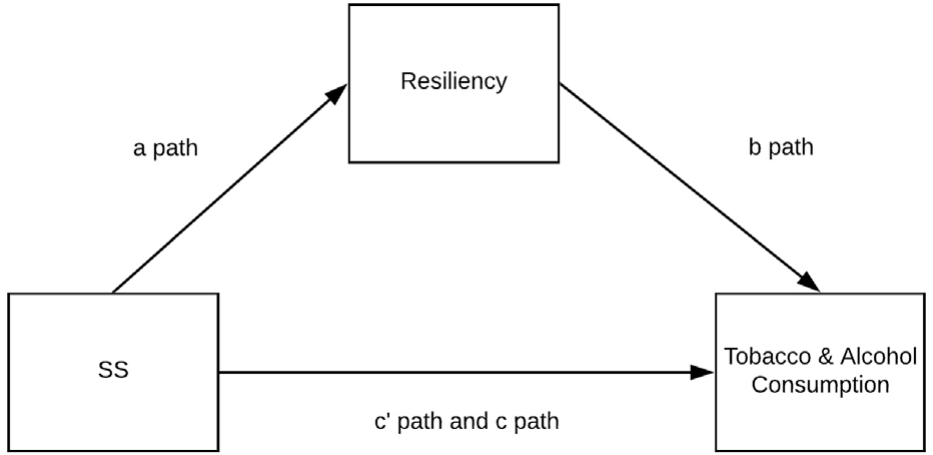
The theoretical model assumed in the study, Poland. Physical activity and dispositional determinants of risky behavior in adolescents, Poland, Pomeranian, 2016

The goal of the current work was to verify the mediation research model presented in Figure 1 in a group of teenage girls and boys with different sporting experiences; thus, the following hypotheses were formed:

Hypothesis 1 SS is a risk factor and resiliency is a protective factor in tobacco and alcohol use.

Hypothesis 2 Resiliency is a mediator of the influence of SS on tobacco and alcohol use.

Hypothesis 3 The strength of the impact of resiliency as a mediator of the influence of SS on tobacco and alcohol use differs depending on gender and whether one participates in sport: the effect on alcohol use is stronger in girls who train, and the effect on tobacco use is stronger in boys who train.

## Methods

### Participants

A total of 649 adolescents from Pomerania in Poland took part in the study. The teenagers were purposively selected from the same age cohort (*M*_*age*_ = 15.93; *SD* = 0.30), to exclude the influence of age on the factors assessed in the study, and were engaged in organised physical activity. Participants were divided into two subgroups: 1) competitive athletes, *n*_*sport*_ = 135 (66 of whom were girls), who trained regularly (hours a week devoted to training: *M* = 14.07; *SD* = 5.16), had valid club licenses, and represented clubs in country-level competitions. The competitive sports athlete group included individuals who trained in team sports (basketball, soccer, volleyball, handball), individual sports (track-and-field, swimming, sport gymnastics), aesthetic disciplines (artistic gymnastics, dance), and martial arts (judo, fencing); and 2) teenagers who participated in physical activity in school (physical education class; PE), *n*_*PE*_ = 514 (of whom 322 were girls). Each athlete also took part in compulsory physical education at school.

Individuals who participated in PE irregularly and those engaged in unorganized physical activity were excluded from the study (18.39% of the initial group). Due to a doctor’s note exempting them from participation in PE classes, 14.57% of the students were excluded from the study. Schools were randomly selected (25 out of 381 general education schools and 10 out of 12 sports schools) from the database of the Pomeranian Office of Education.

### Procedure

The protocol for this study was approved by the Ethics Board for Research at the Institute of Psychology, University of Gdansk, Poland (decision no. 4/2014). Prior to the study, written informed consent was obtained from the participants and their parents, who were also informed that they could discontinue their participation at any time without repercussions. Respondents were given 3 days to complete the questionnaire pack, which was then returned to one of the investigators.

### Measures

#### Risk Behaviors Questionnaire

This tool was developed by Jochimek and Lipowski for the purpose of this study. The goal of the questionnaire was to assess the current risks associated with engaging in tobacco, alcohol or drug use, as well as risky sexual behavior. Competent judges—four psychologists (including a specialist in clinical psychology), three school counselors, two teachers working in a secondary school, and an addiction therapist, gave scores related to the risks associated with a given behavior. For the purposes of this study, two subscales were used:

1) *Tobacco use*—questions regarding having smoked a cigarette or e-cigarette and the frequency of such activities in a week, on which it was possible to score between 0 and 8 points total;
2) *Alcohol use*—having consumed alcohol and/or gotten drunk and the frequency of such activities in a month, on which it was possible to score between 0 and 16 points total.

The questionnaire’s internal consistency, assessed using Cronbach’s *α*, turned out to be satisfactory (*α* = 0.74).

#### The Inventory of Physical Activity Objectives (IPAO)

The IPAO by Lipowski and Zaleski [52] was used to determine the level of involvement in physical activity. The respondent answered questions regarding their involvement in competitive sport (both present and previous), as well as the forms and intensity of their physical activity. Respondents were divided into groups based on this questionnaire.

#### The Brief Sensation Seeking Scale (BSSS-8)

The BSSS-8 by Hoyle et al. [24] was used to determine the participants’ levels of sensation seeking (SS). This scale allows for the prediction of risks associated with the probability of adolescents’ engagement in risky behaviors. The scale is composed of 8 items that participants assess on a 5-level scale (from 1, indicating “strongly disagree,” to 5, indicating “strongly agree”). The results on the four subscales can fall between 2 and 10, and the results of the overall level of sensation seeking can fall between 8 and 40. The subscales are as follows:

1) *Thrill and Adventure Seeking*—defined as the willingness to participate in physical activity related to speed, danger, novelty and counteracting gravity, such as parachuting, diving, or bungee jumping;
2) *Experience Seeking*—a tendency to seek new experiences related to travel, music, art, or a non-conformist lifestyle with like-minded people;
3) *Disinhibition*—the need to look for a way to relieve tension, without inhibition and embarrassment, through various social activities;
4) *Boredom Susceptibility*—is expressed through a reaction of tension and reluctance in a situation where it is necessary to engage in activities that require repetition and routine work, or while being with predictable people.

The higher the results on the subscales, the higher the sensation seeking. Adolescents with a higher level of sensation-seeking, compared to those with a lower intensity, engage in risky behaviors to a greater extent and tend to assess their actions as less threatening. The internal consistency of the original version of the questionnaire was confirmed, and its Cronbach’s *α* was 0.76 [24]; the internal consistency of the Polish version of the BSSS-8 was satisfactory, with Cronbach’s *α* equal to 0.80.

#### Resiliency Assessment Scale for Children and Adolescents (SPP-18)

The SPP-18 by Ogińska-Bulik and Juczyński [43] was used to evaluate levels of resiliency as a personality trait. SPP-18 also allows for the description of the functioning of the adolescent using the four dimensions of resilience by subscales: *optimistic attitude and energy, perseverance and determination to act, sense of humor and openness to new experiences, personal competences and tolerance of negative affect.* The reliability of the instrument, determined by its authors on the basis of Cronbach’s alpha, was 0.82 for the whole scale. Respondents assess 18 items on a 5-point scale (from 0, “strongly disagree,” to 4, “strongly agree”). The more points there are, the higher the respondent’s resiliency. Moreover, the raw scores can be recalculated into a sten scale (where a score of 1–4 indicates low, 5–6 average, and 7–10 high resiliency), and mean scores indicating the importance of particular subscales can also be calculated.

### Statistical Analysis

Using analysis of variance (ANOVA) with a *post hoc* Tukey analysis for different N values, we initially examined differences between athlete and non-athlete girls and boys in terms of drinking, sensation seeking, and resiliency. In the next step, to test the hypothesis that the impact of sensation seeking on tobacco and alcohol use is mediated by resiliency, we performed mediation analyses. Using the PROCESS bootstrapping macro [53], we entered *sensation seeking* as the predictor, *resiliency* as the hypothesized mediator, and *tobacco* and *alcohol use* as separate dependent variables (we applied analysis model 4 in the PROCESS bootstrapping macro). We made this mediation separately for boys and girls, athletes and the PE group, and then separately for each group.

## Results

To evaluate tobacco and alcohol use, sensation seeking, and resiliency levels with regard to gender and sport, we performed a two-way ANOVA (2: gender × 2: sport; Table 1). No statistically significant gender- or physical activity-related differences were observed on the tobacco or alcohol use scales; on average, 40.42% of young people had smoked cigarettes and 9.19% had used e-cigarettes, while alcohol was used by 71.84% of respondents and as many as 32.51% had gotten drunk at least once. Boys scored higher (*M* = 51.17, *SD* = 8.90) than girls (*M* = 48.54, *SD* = 9.84) in terms of resiliency (*p* = 0.049). The athlete group had higher SS (*M*_athletes_ = 28.87, *SD*_athletes_ = 6.06, *M*_PE_ = 26.77, *SD*_PE_ = 6.37, *p* = 0.012) and resiliency (*M*_athletes_ = 52.47, *SD*_athletes_ = 8.52, *M*_PE_ = 47.24, *SD*_PE_ = 10.23, *p* < 0.001) scores. No gender x sport effect was observed for SS. However, male athletes scored higher on resiliency compared with the male PE (*p* < 0.001), female PE (*p* < 0.001), and female athlete (*p* = 0.019) groups (Table 1).

**TABLE 1:** Differences in tobacco and alcohol use, sensation seeking, and resiliency with respect to gender and sport (ANOVA), Poland. Physical activity and dispositional determinants of risky behavior in adolescents, Poland, Pomeranian, 2016.

	Female athletes (*n* = 66)	Female PE (*n* = 322)	Male athletes (*n* = 69)	Male PE (*n* = 192)	Gender		Sport		Gender x sport	
	*M* (*SD*)	*M* (*SD*)	*M* (*SD*)	M (SD)	*F*	(p*)	*F*	(*p*)	*F*	(*p*)
Outcomes:										
Tobacco use	0.77 (1.32)	0.95 (1.56)	1.01 (1.54)	0.77 (1.39)	0.03		0.04		2.12	
Alcohol use	2.08 (1.96)	2.01 (2.33)	2.17 (2.10)	1.86 (2.47)	0.05		3.78		1.52	
Predictor:										
Sensation seeking	28.83 (6.68)	27.46 (6.50)	28.91 (5.43)	26.07 (6.24)	1.13		**11.69**	**(.001)**	1.42	
Mediator:										
Resiliency	49.92 (8.75)	47.15 (10.93)	55.01 (1.85)	47.32 (8.28)	**7.22**	**(0.007)**	**28.52**	**(<0.001)**	**6.28**	**(0.012)**

*p-values are only provided for statistically significant results.

Bold values indicate that the result is statistically significant.

Further on, the multivariate regression analysis of resiliency and sensation seeking as predictors of tobacco and alcohol use with respect to gender and physical activity were performed (see Table 2).

**TABLE 2:** Resiliency and sensation seeking as predictors of tobacco and alcohol use with respect to gender and sport (multivariate regression analysis), Poland. Physical activity and dispositional determinants of risky behavior in adolescents, Poland, Pomeranian, 2016.

	Alcohol use β (*p*)				Tobacco use β (*p*)			
	Sport		PE		Sport		PE	
	♀	♂	♀	♂	♀	♂	♀	♂
Resiliency	−0.18 (0.136)	−0.11 (0.388)	−0.19 (0.001)	0.02 (0.762)	−0.27 (0.028)	−0.30 (0.019)	−0.16 (0.003)	0.12 (0.096)
Sensation seeking	0.40 (0.001)	0.32 (0.015)	0.25 (0.000)	0.17 (0.024)	0.33 (0.007)	0.33 (0.010)	0.33 (0.000)	0.18 (0.011)
Summary of the regression *F (p) R* ^*2*^	5.97	3.11	13.86	2.84	5.33	4.63	20.39	5.63
	(0.004)	(0.051)	(0.000)	(0.061)	(0.007)	(0.013)	(0.000)	(0.004)
	0.08	0.09	0.07	0.03	0.15	0.12	0.11	0.06

The regression analysis showed that resiliency was a significant predictor of alcohol use in case of girls who take part in PE, and for tobacco use in both the female athlete and PE groups. Furthermore, sensation seeking was also significant predictor for alcohol and tobacco use in female and PE group. In the case of boys, resiliency was a significant predictor only for tobacco use in the male athlete group. Sensation seeking was a significant predictor for alcohol use, as well as for tobacco use in the male and PE group. Resiliency seems to be a protective factor against alcohol and tobacco use, whereas sensation seeking was a risk factor, thus, Hypothesis 1 was confirmed.

Next, to verify the protective role of resiliency as a mediator of the relationship between sensation seeking and tobacco and alcohol use in the context of gender and physical activity, a series of mediation models was run. The analysis indicated that the effect of sensation seeking on tobacco and alcohol use through the mediating effect of resiliency was significant for the female PE group and the male athlete group.

**FIGURE 2:**
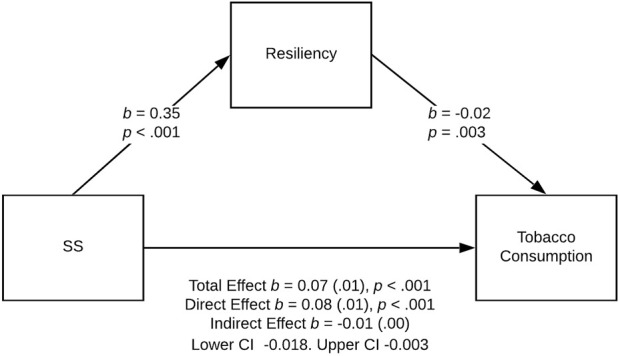
Relationship between sensation seeking and tobacco use partially mediated by resiliency in the female PE group, Poland. Note: Standard errors are provided in parentheses.Physical activity and dispositional determinants of risky behavior in adolescents, Poland, Pomeranian, 2016

**FIGURE 3:**
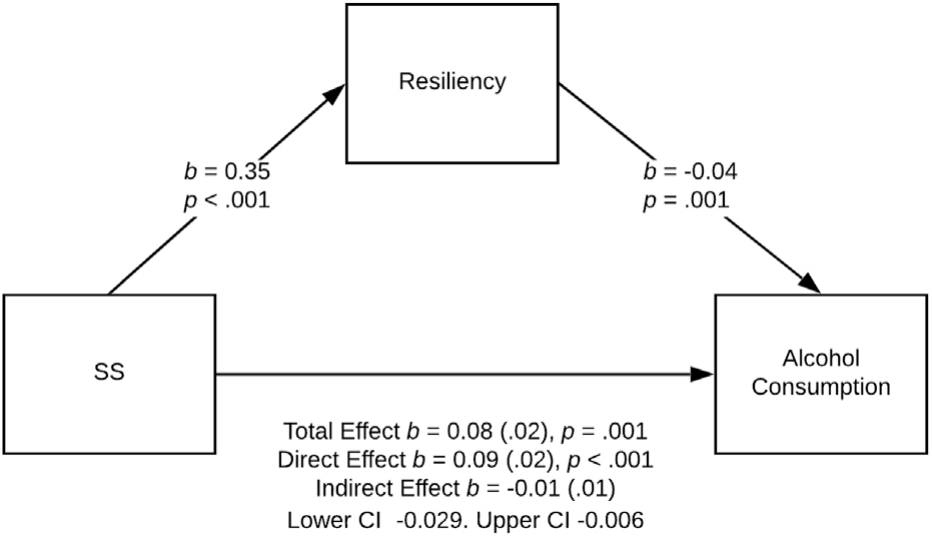
Relationship between sensation seeking and alcohol use partially mediated by resiliency in the female PE group, Poland. Note: Standard errors are provided in parentheses. Physical activity and dispositional determinants of risky behavior in adolescents, Poland, Pomeranian, 2016

**FIGURE 4:**
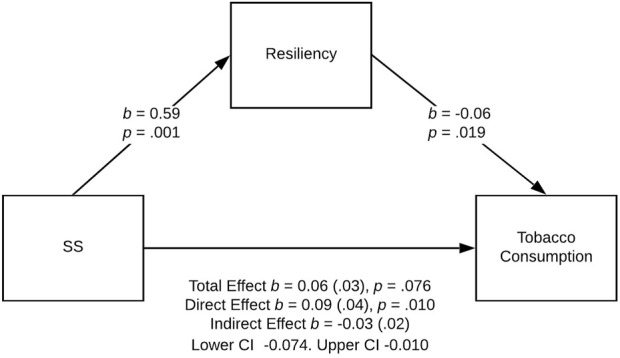
Relationship between sensation seeking and tobacco use fully mediated by resiliency in the male athlete group, Poland. Note: Standard errors are provided in parentheses. Physical activity and dispositional determinants of risky behavior in adolescents, Poland, Pomeranian, 2016

Three models were statistically significant: the effect of sensation seeking on tobacco and alcohol use was partially mediated by resiliency in the female PE group (Figures 2, 3), and the effect of sensation seeking on tobacco use was fully mediated by resiliency in the male athlete group (Figure 4).

In the case of tobacco use in the female PE group, the bootstrap confidence interval for the indirect effect (*b* = −0.02) based on 5,000 bootstrap samples was entirely under zero (−0.018 to −0.003; see Figure 2). Thus, we found a stronger direct effect of sensation seeking on alcohol use (*b* = 0.08, *SE* = 0.01, *t* (319) = 6.14, *p <* 0.001, 95% CI for *b* = [0.054, 0.105]). The *R* ^2^ for the model was 0.11; *F* (2, 319) = 20.39, *p* < 0.001.

For alcohol use in the female PE group, the bootstrap confidence interval for the indirect effect (*b* = −0.021) based on 5,000 bootstrap samples was entirely under zero (−0.029 to −0.006; see Figure 3). Thus, we found a stronger direct effect of sensation seeking on alcohol use (*b* = 0.09, *SE* = 0.02, *t* (319) = 4.59, *p <* 0.001, 95% CI for *b* = [0.052, 0.129]). The *R* ^2^ for the model was .08; *F* (2, 319) = 13.86, *p* < 0.001.

Finally, the effect of sensation seeking on tobacco use was fully mediated by resiliency in the male athlete group (Figure 4). The bootstrap confidence interval for the indirect effect (*b* = −0.03) based on 5,000 bootstrap samples was entirely under zero (−0.074 to −0.010; see Figure 4). Thus, we found a stronger direct effect of sensation seeking on alcohol use (*b* = 0.09, *SE* = 0.04, *t* (66) = 2.65, *p =* 0.010, 95% CI for *b* = [0.023, 0.165]). The *R* ^2^ for the model was 0.12; *F* (2, 66) = 4.63, *p* = 0.013.

## Discussion

Our research showed that 16-year-old athletes were characterized by higher levels of sensation seeking and resiliency. Studies on factors influencing the levels of physical activity undertaken—from moderate to intense—have shown that higher levels of sensation seeking and the tendency to take risks are associated with undertaking physical activity [54, 55]. Moreover, the higher level of resiliency in the group of athletes compared to the group that only took part in physical education classes is in line with previous research about resiliency in groups with high levels of physical activity [48–50]. It is worth mentioning that resiliency levels in the male athlete group were higher than those in the other research groups, and according to the normal values, this group had particularly high levels of this personality trait [43]. It is thus possible to conclude that the male athlete group is characterized by the greatest elasticity and ability to act in distressing situations and that they are more likely to treat difficult situations as challenges. Individuals with higher levels of resiliency are more persistent in working towards their goals [43]. In the developing area of research on resiliency in sport, it is emphasized that there are a different number of stressors in an athlete’s life compared to a non-athlete due to the associated training, pressure, and adversities (e.g., competing, injuries) [56]. How athletes cognitively interpret events, especially failures, is very important; failures may be treated as learning opportunities, which is characteristic of people with higher resiliency. In line with this idea, athletes should be characterized by a positive personality (openness to experience, diligence, creativity, extroversion, emotional stability, optimism, and proactively coping with stress), motivation, self-confidence, focused attention, and perceived social support [56, 57]. These elements form mental resiliency, according to the understanding of Polish researchers [43]. Bell and Suggs [58] and Bell [59] suggest that the features that constitute resiliency can be developed, similar to muscle tissue, and sport is an opportunity to train an indomitable fighting spirit, which is often associated with the need to overcome difficulties. In line with this idea, sport is one activity that fosters the development of personality features responsible for elastic adaptation to adverse events. This thesis is supported by the research of Litwic-Kaminska and Izdebski [50], who have shown that the higher the level of resiliency in athletes, the more positive the light in which they view stressors, and the more likely they are to have a coping style that focuses on the task.

The youth who took part in this research engaged in risky behaviors associated with experimenting with smoking cigarettes and drinking alcohol, and the groups did not differ between each other in this regard. However, individual differences in personality profiles, gender, and levels of engagement in physical activity differentiated the mechanism of engagement in risky behaviors. Resiliency was shown to be a protective factor against smoking cigarettes and drinking alcohol—visible in regard to girls who participated in PE classes and boys who trained (only in the case of tobacco use). Thus, the second and third hypotheses can only be partially accepted.

One of the goals of physical education at schools is to foster the development of pro-health behaviors, social competences, and the personalities of the students. It is worth considering that the protective effect of resiliency as a mediator of the influence of sensation seeking on tobacco and alcohol use was only observed in girls who participated in physical education classes. This means that in the case of boys, one needs to pay special attention to activities that develop protective factors such as persistence and determination, an optimistic attitude, and social competences, which are the components of resiliency.

It should be mentioned that a higher influence of sensation seeking on resiliency was noted in the male athlete group, and in this case, resiliency was a factor protecting against tobacco use. This result indicates that it might be possible to focus an adolescent’s energy on sports training, through which they can fulfil their need for success and seek strong sensations in a socially acceptable way [60]. It is surprising that resiliency did not constitute a protective factor against tobacco and alcohol use in female athletes.

The combination of developmental psychology and sports psychology in the area of analyzing factors that protect teenagers from the risk of experimenting with smoking and alcohol use is a strength of the current study. There are a limited number of studies concerned with this topic in athletes who are just beginning intensive training. Moreover, a representative group of teenagers took part in this study. However, in future studies, the athlete group should be larger, which will eliminate the possible effect of differences in sample sizes on the results.

### Conclusion and Practical Implications

This study highlights the same level of engaging in risky behaviors associated with experimenting with smoking cigarettes and drinking alcohol by both young athletes and non-athletes. The factor that increases the risk of resorting to alcohol and tobacco use seems to be a high level of sensation seeking. Conversely, resiliency seems to protect youth against undertaking risky behaviors. Moreover, the findings of this study indicate that engaging in sport fosters resiliency. Young athletes, especially males, showed a higher level of resiliency than their peers not engaged in professional sport.

Adolescence is a particularly vulnerable time in a lifetime when many teenagers experience different health issues. Therefore, it seems extremely important to strengthen their competences to deal with the different life difficulties and developmental challenges. According to the above, one of the goals of physical education at schools should be promoting the development of pro-health behaviors, as well as social competences and youth abilities to manage with stress [61, 62]. The growing body of research suggest that sport is one of the activities that foster the development of personality features responsible for adaptation to the challenges, such as resiliency. Furthermore, our findings suggest that due to individual differences in personality profiles, gender, and levels of engagement in physical activity, the mechanisms of engagement in risky behaviors are different. Risky behaviors typical for adolescents such as smoking cigarettes and drinking alcohol can be very harmful for adolescents health, and can affect not only the deterioration in their cognitive functioning, school, relationships with loved ones, but also cause health problems—both in terms of physical as well as mental health [25, 41, 47, 62]. It is known that the use of alcohol, other drugs, and smoking cigarettes are strongly associated with the mortality and morbidity among adolescents [63]. Therefore, it is worth paying attention to the role of resiliency and engagement in sport as important factors against risky behaviors (in our study this is visible especially in regard to girls who participated in PE classes and boys who trained), thus affecting the health of adolescents. Attention should be paid to creating appropriate risk behaviors prevention programs at school, as well as using sports activities, encouraging teenagers to engage in a variety of physical activity, thus increasing their personal resources and reducing the risk of health problems.
